# The impact of the use of masks on trait judgments and face recognition

**DOI:** 10.3758/s13421-023-01495-3

**Published:** 2023-11-27

**Authors:** Raquel Pinto, Pedro B. Albuquerque

**Affiliations:** School of Psychology, University of Minho (Portugal), Campus de Gualtar, 4710-057 Braga, Portugal

**Keywords:** Face recognition, Trait judgments, Face masks, Memory

## Abstract

Although effective in reducing virus transmission, face masks might compromise face recognition and trait judgments. With this study, we aimed to observe the influence of masks on face recognition and trait judgments—more specifically, in trustworthiness, dominance, and distinctiveness judgments. Also, we wanted to observe the possible influence of trait judgments on facial recognition for masked and unmasked faces, which has never been done before. For that, we conducted an online study where 140 participants observed and made trait judgments of masked and unmasked faces in a within-subjects design. After a distractive task, participants performed a recognition memory test. As expected, we observed a better recognition of faces shown without a mask during the study phase, which allowed the holistic processing of the faces. The worst performance was found for faces encoded with a mask but tested without it, occurring simultaneity disruption in holistic face processing and the violation of the encoding specificity principle. Regarding the trait judgments, unmasked faces were considered more distinctive, and masked faces were considered more trustworthy. More interestingly, we can conclude that facial distinctiveness predicts face recognition, regardless of mask use. In contrast, dominance judgments only predicted face recognition when faces were presented without a mask. When faces were exposed with masks, trustworthiness overrides dominance, becoming more critical to recognizing faces. We can interpret these results from an evolutionary perspective.

## Introduction

One of the public health guidelines that the World Health Organization (WHO) recommended as a response to prevent the spread of COVID-19 was the use of face masks (WHO, 2020). Although the world is returning to normality, with face masks no longer mandatory in some countries, as in the case of Portugal (Decree-Law 30-E/2022, 2022), many people continue to use them to prevent infections, particularly in public places as public transport and health institutions.

The use of face masks, despite being effective in reducing virus transmission (Howard et al., 2021), impairs face recognition because the face masks cover about 60% to 70% of the face area that is relevant for the identification of a person (e.g., Tsao & Livingstone, 2008)—namely, the nose, mouth, and chin. The impact of the use of masks in face recognition is not surprising because faces seem to be processed holistically (Farah et al., 1998; Logan et al., 2017; Meltzer & Bartlett, 2019; Taubert et al., 2011), which means that faces are perceived as a whole rather than a single combination of each component (e.g., eyes, nose, mouth). So, when a face is occluded or covered by a mask, sunglasses, hat, beard, scarf, or a religious veil, it is impossible to perform the holistic processing of the face (e.g., Davies & Flin, 1984; Hockley et al., 1999; Mansour et al., 2017; Nguyen & Pezdek, 2017; Righi et al., 2012), which appears to deteriorate its recognition (e.g., McKelvie, 1976).

Freud et al. (2020) corroborated these results and showed that masks appeared to impair face recognition by disrupting holistic processing. The authors instructed participants to complete the Cambridge Face Memory Test (Duchaine & Nakayama, 2006) and observed that face masks impaired identity recognition. This result occurred regardless of the mask being present when a face is encountered at encoding or during a recognition test. In particular, the authors (Freud et al., 2020) found that holistic processing was disrupted for faces with masks, as suggested by a reduced inversion effect. So, when faces are presented upside-down, they take longer to process, suggesting that disrupting the usual way we perceive faces (i.e., upright) affects their holistic processing (e.g., Taubert et al., 2011). Nevertheless, when masked faces are presented upside-down, they do not take long to process since holistic processing was already disrupted.

The impact of the use of the mask on face recognition is particularly important in eyewitness testimony, namely on lineup person identification (e.g., Manley et al., 2019, Exp. 2). The authors found that the recognition performance was better when participants observed an unmasked face at the study phase (i.e., encoding phase) and with a full-face lineup (unmasked face at the test phase). However, their worse performance occurred when participants observed a face with a ski mask during the study phase and a full-face lineup. So, results showed that matching the conditions (i.e., the use of a ski mask) in the study and test phase results in a better eyewitness identification performance. The authors (Manley et al., 2019) explained these results based on the transfer-appropriate processing framework, which proposes that the performance on a memory test is higher when the processes activated at retrieval match those at encoding—encoding specificity principle (e.g., Morris et al., 1977). Therefore, this principle suggests that the impact of a mask on facial recognition likely also depends on the relationship between study and test conditions (i.e., if the conditions between the study and test phase match or not).

The impact of the disruption in holistic face processing and the violation of the encoding specificity principle on face recognition was also shown in a study implemented by Guerra et al. (2022). In this study, better face recognition occurred when faces were presented without a mask during the study and the test phase (i.e., at the congruent unmasked condition). As in Manley et al.’ (2019) study, worse recognition performance was observed when faces were presented with a mask during the study phase but tested without it (i.e., masked–unmasked incongruent condition). The authors explained this worse performance by the disruption in holistic face processing and the violation of the encoding specificity principle (Guerra et al., 2022). In the condition where faces were presented without a mask in the study phase but with a mask in the test phase, it also occurred the violation of the encoding specificity principle (i.e., the conditions between the study and test phases did not match). Still, the performance did not suffer a decay to the same extent. The holistic processing that occurred during the study phase, where the faces were presented without masks, likely protected the face recognition performance (Guerra et al., 2022).

Besides face recognition, it was observed that face masks also impair judgments of friendliness or attractiveness (Goldstein & Brockmole, 2017; Patel et al., 2020) and emotional identification (Carbon, 2020; Eisenbarth & Alpers, 2011). It was observed, for example, that faces with emotions whose discrimination depends heavily on mouth configuration, such as sadness, happiness, and anger, were often misinterpreted as neutral faces when covered by a mask (Carbon, 2020). Since face masks reduce the accuracy and confidence in emotion recognition (Carbon, 2020), it was hypothesized that masks might also affect trait judgments. For example, Patel et al. (2020) observed that faces considered average or unattractive at baseline were judged more attractive when wearing masks.

The impact that faces masks may have on social perception and cohesion has been studied more recently (Oldmeadow & Koch, 2021). For example, surgical masked faces were rated more trustworthy than unmasked ones (Olivera-La Rosa et al., 2020). The authors speculated that “the internalized social norm of wearing a mask is suppressing any automatic mistrust due to not seeing the whole face” (Olivera-La Rosa et al., 2020, p. 5). The perceptual process supported another explanation. The low trustworthiness was based on cues that were covered by the masks, and for this reason, trustworthiness judgments increased. Higher trustworthiness judgments were also previously observed when the nose or mouth was covered by a black rectangle (Santos & Young, 2011).

The use of masks was also associated with an increased rating of attractiveness and had a small effect on dominance/competence (Oldmeadow & Koch, 2021). This small effect of the masks on dominance judgments was explained with evidence that suggests these judgments rely primarily on structural cues such as eyes-to-eyebrow distance and width-to-height ratio (Costa et al., 2017; Vernon et al., 2014), which are not affected by the use of a mask. It remains unclear why using face masks increased perceived trustworthiness and attractiveness and whether this is due primarily to positive social norms for mask-wearing or perceptual processes.

Interestingly, the trustworthiness and attractiveness judgments were affected by the inversion of the faces (i.e., when the holistic processing is disrupted) but not the distinctiveness of the faces. One possible explanation is that distinctiveness judgments are made mainly based on particular facial features, which could still be perceived in upside-down faces. So, specific local aspects of the faces influence participants’ distinctiveness judgments, explaining the absence of a significant effect of inversion (Santos & Young, 2008).

Therefore, with this experiment, we wanted to observe the influence of masks on face recognition and trait judgments: trustworthiness, dominance, and distinctiveness. Our first hypothesis poses a worse recognition memory for masked faces regardless of the mask being presented at the study or test phase (Freud et al., 2020; Guerra et al., 2022). In our second hypothesis, we anticipated worse recognition memory performance for faces wearing masks at encoding but not at the test phase (Freud et al., 2020; Guerra et al., 2022; Manley et al., 2019). Concerning the judgments, in our third hypothesis, we anticipated that faces with masks would be considered more trustworthy while unmasked faces would be perceived as more distinctive (Oldmeadow & Koch, 2021; Olivera-La Rosa et al., 2020). Lastly, in our fourth hypothesis, we did not expect differences between masked and unmasked faces regarding the dominance judgments (Oldmeadow & Koch, 2021).

Finally, we wanted to observe the possible influence of trait judgments on facial recognition for masked and unmasked faces, which has never been done before. It was previously observed that trait judgments of a face yield better subsequent recognition memory than physical feature assessments (e.g., Mueller et al., 1978). This result was explained based on the levels-of-processing framework (Craik & Lockhart, 1972), in which the trait judgment-encoding task promotes a deeper, more elaborate processing of the face (Wells & Hryciw, 1984). So, our goal was to observe how these trait judgments can affect the facial recognition of masked and unmasked faces.

## Method

### Participants

This sample integrated 140 undergraduate students (129 females) aged between 18 and 36 years (*M*_age_ = 20.21 years, *SD* = 2.65). The sample size was calculated based on the study of Guerra et al. (2022), which aimed to observe the impact of the use of masks on face recognition. We considered the same sample size of 140 participants since the same stimuli and experimental manipulation were applied, even considering that the effect size to the relevant interaction on such experiment was η_p_^2^ = .28. The importance of comparing the results of the present study with the former one (Guerra et al., 2022) was the main reason to opt for a such number of participants. Written consent was obtained from all participants who received course credits for their participation. The local Ethics Committee approved this experiment.

### Materials

The faces used in this experiment were selected from the free access “The Face Chicago Database” (Ma et al., 2015). We recurred to 48 neutral Caucasian faces (24 male and 24 female). Several criteria to their selection were applied: estimated age between 18 and 26 years old (*M* = 23.82, *SD* = 1.81) and masculinity and femininity judgment above 98%. Also, we opted for neutral facial expressions, and for this, we selected faces whose emotional expressions were evaluated below 3.5 on a 7-point Likert scale (1 = *not at all*; 7 = *extremely*). A face evaluated with 1 means that the face does not look, for example, happy at all, while a face considered with 7 means that the face seems extremely happy. The average evaluation for emotional expressions of the faces used in this experiment was: afraid = 2.04; angry = 2.37; disgusted = 2.10; happy = 2.28; sad = 2 .61; and surprised = 1.66.

Every photo was duplicated, and one of the items was then manipulated with Adobe Photoshop CC (Version 2.2) by superimposing a surgical mask on the original photo, a technique usually used in other studies (e.g., Carragher & Hancock, 2020; Guerra et al., 2022). All 48 faces had two versions: unmasked (original photo) and masked (manipulated photo). The faces manipulated are available in the OSF platform (https://osf.io/5mjkv/?view_only=06e45ed1d0064cb0b86a9a96f2675899). The experimental procedure was created using Qualtrics XM (Qualtrics, 2021).

### Procedure

**Fig. 1:**
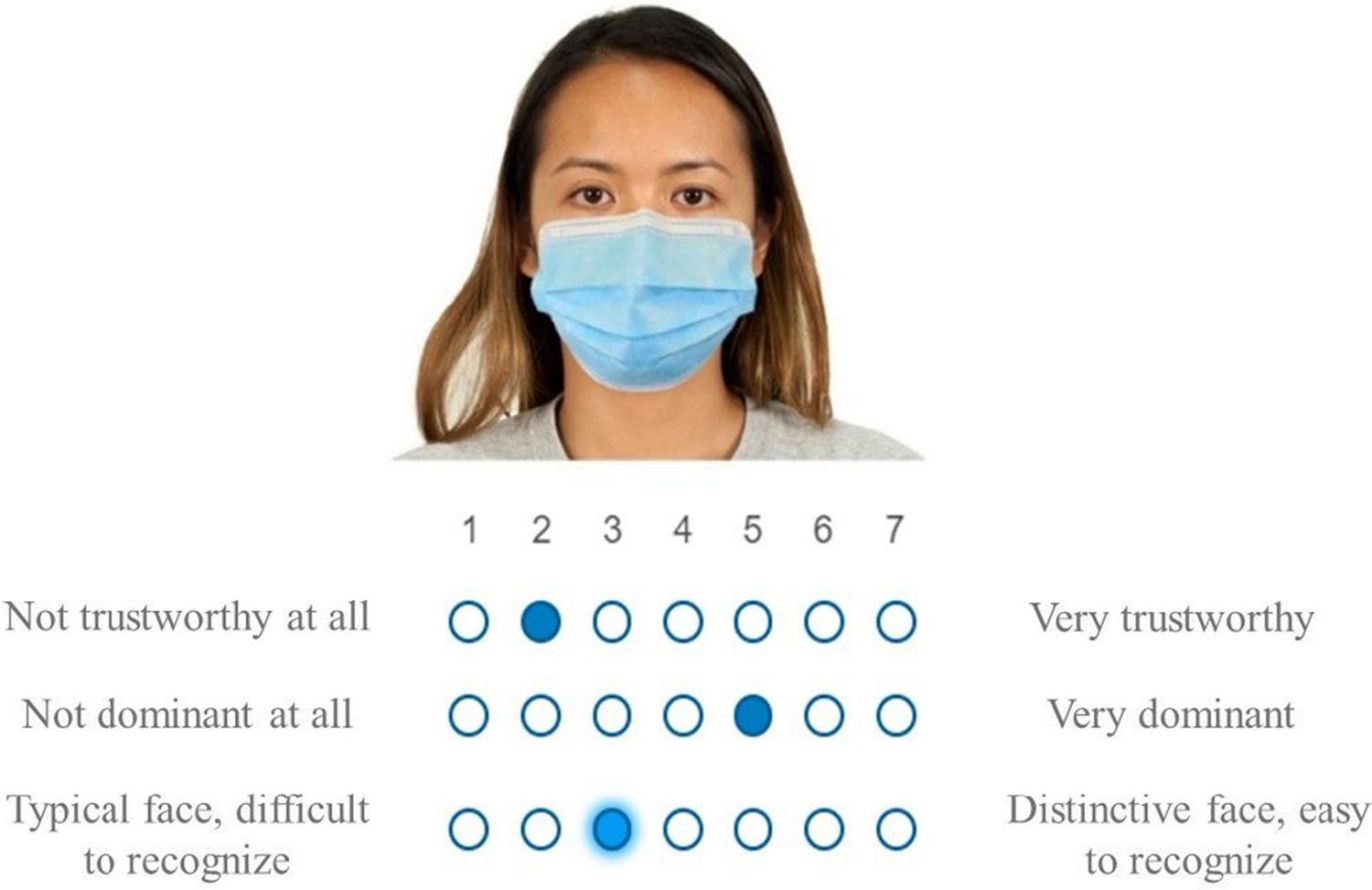
Illustrative scheme of stimuli presented in the study phase

The study was conducted entirely online. Participants were presented with informed consent and a sociodemographic questionnaire to complete at the beginning of the procedure. The main procedure included two phases: a study and a test phase. In the study phase, 32 faces were randomly presented, matched by sex (half depicting masked faces and the other half unmasked faces). Participants were instructed to pay attention to the masked and unmasked faces presented, given that later they would have a recognition memory test. Additionally, they were asked to perform judgments on each face. The stimuli were counterbalanced across participants so that each face was presented with a mask for half of the participants and without a mask for the rest. Below each face were shown three different scales to perform the judgments, where participants were asked to evaluate the face presented regarding their distinctiveness, trustworthiness, and dominance. The order of the three scales was presented randomly for each participant (see Fig. 1 for an example).

Regarding the trustworthiness rating, participants were instructed to “observe each face and indicate how trustworthy each face is to you, that is, to what extent you could trust this person.” The 7-point rating scale was also described, indicating that 1 corresponded to *not trustworthy at all* and 7 to *very trustworthy*.

Considering the dominance rating, participants were instructed to rate each face’s dominance using a 7-point rating scale, where 1 corresponded to *not dominant at all* and 7 to *very dominant*. Dominance is defined as an individual’s ability to be influential, respected, and often a leader. Lastly, in the distinctiveness-rating instructions, participants were instructed to rate how distinctive each face was to them, where 1 corresponded to *typical face, difficult to recognize* and 7 to *distinctive face, easy to recognize*. Each face was displayed for 20 seconds with a blank screen interval of 2 seconds between them.

Following the study phase, where participants observed and made judgments about the faces, participants performed a distractive task for 3 minutes. They had to type as many category exemplars as possible for 30 seconds (e.g., body parts, European countries, fruits, colors, furniture items). The categories were presented individually, with a 5-second interval between them.

Thirty-two images were presented in the test phase, 16 as targets and the other 16 as distractors. The distractors were balanced according to the presence or absence of a mask. Of the 16 targets, half were previously presented with masks, and the other half were unmasked. In sum, four faces appeared with a mask in both moments (masked-masked); four faces were presented unmasked in the two phases (unmasked–unmasked); four faces appeared in the study phase without a mask but in the test phase with a superimposed mask (unmasked-masked); and, finally, four faces were exposed in the study phase with a mask but in the test phase without it (masked–unmasked). The stimuli were randomly presented, and for each stimulus, two response options were displayed: “Yes, this person was presented before” and “No, this person was not presented before.” Using their computer mouse, participants judged whether that person had been presented before or whether it was the first time they had seen it. The procedure took approximately 40 minutes to complete.

### Design and analyses

A 2 (face at study: masked vs. unmasked) × 2 (face at test: masked vs. unmasked) repeated-measures ANOVA was performed to explore the effect of a mask on face recognition. Face recognition performance was measured through the sensitivity index or *d*-prime. A *d*-prime (*d′*) score [*z* (Hits) − z (False Alarms)] was calculated for the recognition memory results on each condition. We also calculated the response bias (*c*) score [− (*z* (Hits) + *z* (False Alarms))/2]. Hits refer to “yes” responses to the faces that were presented in the study phase (correct “yes” answer), and false alarms refer to “yes” responses to faces that were not shown in the study phase (incorrect “yes” responses). Additionally, to observe if any differences exist in face recognition when the presence or absence of mask at the two phases (i.e., study and test) were congruent (masked–masked, unmasked–unmasked) and incongruent (masked–unmasked, unmasked–masked), we applied a paired-sample *t* test.

To observe the effect of a mask on trait judgments, we performed a paired-samples *t* test for each of the variables evaluated: trustworthiness, distinctiveness, and dominance, in which we compared the assessments made for faces with mask and unmasked faces. The dependent variables were the ratings of trustworthiness, distinctiveness, and dominance given to face images. This data is available in the OSF platform (https://osf.io/5mjkv/?view_only=06e45ed1d0064cb0b86a9a96f2675899).

As in previous studies (Oldmeadow & Koch, 2021), these ratings collapsed across face sex because sex was not a variable of interest in this study. Even though it was observed that sex is relevant to trustworthiness judgments (e.g., Mattarozzi et al., 2015), these effects depend on perceiver gender. Since gender was not the focus of the current paper, and for this reason, it was not evaluated, we have collapsed across face sex to simplify the interpretation of main effects and interactions.

Finally, four multiple regression analyses were carried out to observe the possible influence of trait judgments on facial recognition, determining which variables (i.e., trustworthiness, distinctiveness, and dominance) can influence and predict face recognition. Multiple regressions were performed for each condition (i.e., masked–masked, unmasked–unmasked, masked–unmasked, and unmasked–mask) to understand if a particular variable only influences face recognition under a specific condition (e.g., faces without masks). This influence can cease under other conditions—namely, when faces are presented with masks. The software used for all these data analyses was JASP 0.15 (JASP Team, 2021).

## Results

### Effect of masks on face recognition

Regarding the effect of a mask on face recognition, a significant main effect of the Face at Study was found, *F*(1, 139) = 48.14, *p* < .001, η_p_^2^ = .26, revealing a better recognition for unmasked faces (*M* = 1.84; *SE* = .06) compared with masked faces at study (*M* = 1.35; *SE* = .06). A main effect of the Face at Test was also found, *F*(1, 139) = 33.50, *p* < .001, η_p_^2^ = .19, showing a better recognition for faces masked at test (*M* = 1.72; *SE* = .05) than unmasked faces (*M* = 1.47; *SE* = .05). Additionally, we found a significant interaction, *F*(1, 139) = 86.96, *p* < .001, η_p_^2^ = .39.

**Fig. 2:**
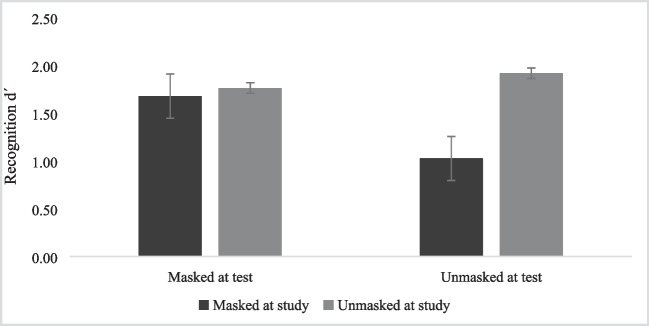
Mean recognition d′ scores for masked and unmasked faces at the study and test phase. Note. Error bars represent standard errors

To explore this interaction and observe possible differences between the four groups (masked at the study and masked at the test; unmasked at the study and unmasked at the test; masked at the study and unmasked at the test; and unmasked at the study and masked at the test), we conducted multiple comparisons, applying the Bonferroni correction. It was observed a significantly better recognition of faces presented without a mask in both phases (all *p*s < .05) compared to other conditions. Also, it was observed a significantly worse recognition of faces presented with a mask in the study phase and without a mask in the test phase (all *p*s < .001) compared to all the other conditions (see Fig. 2). So, we can conclude that face recognition was more impaired when faces were presented with masks in the study phase and without a mask in the test phase.

**Fig. 3:**
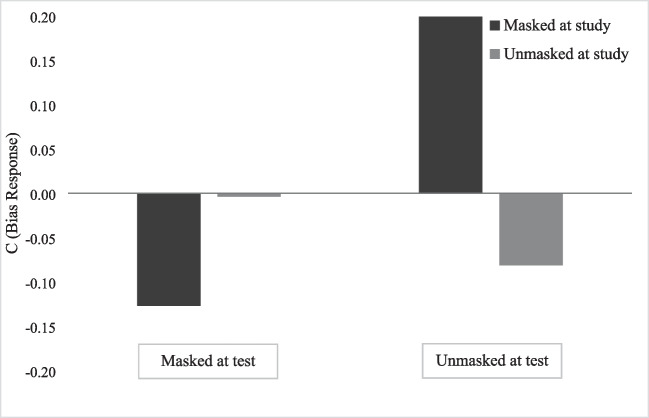
Mean recognition c scores for masked and unmasked faces at the study and test phase

Considering the analysis of response bias ©, a significant main effect of the face at study was found, *F*(1, 139) = 5.85, *p* = .02, η_p_^2^ = .04, revealing more conservative criteria for the recognition of masked faces (*M* = .04; *SE* = .03) compared to unmasked faces at study (*M* = −.04; *SE* = .03). A significant main effect of the face at test was also found, *F*(1, 139 = 33.63, *p* ≤ .001, η_p_^2^ = .20, showing a higher *c* value for unmasked faces (*M* = .06; *SE* = .03) than for masked faces at test (*M* = −.07; *SE* = .03). Finally, we found a significant interaction between both factors, *F*(1, 139) = 86.92, *p* < .001, η_p_^2^ = .39. Post hoc tests with Bonferroni correction revealed a significantly higher *c* value for the condition where the faces were presented with masks at study and unmasked at test (all *p*s < .001), expressing a more conservative answer at the recognition of such faces (see Fig. 3).

### Effect of masks on trait judgments

Regarding the effect of a mask on trait judgments, we observe that faces without a mask (*M* = 4.21, *SE* = .09) were considered more distinctive than masked faces (*M* = 3.60, *SE* = .08), *t*(47) = 6.90, *p* < .001, Cohen *d* = 0.99, 95% CI [0.65, 1.34]. However, masked faces were considered more trustworthy (*M* = 3.96, *SE* = .08) than faces without a mask (*M* = 3.51, *SE* = .09), *t*(47) = 6.15, *p* < .001, Cohen *d* = 0.89, 95% CI [0.55, 1.22]. No differences were observed between masked (*M* = 3.46, *SE* = .09) and unmasked faces (*M* = 3.58 *SE* = .10) regarding dominance judgments (*p* > .05).

### Effect of trait judgments on face recognition

Finally, multiple regressions were carried out to observe the possible influence of trait judgments on facial recognition and to determine whether this effect occurs similarly in all the conditions (i.e., with masked and unmasked faces). When faces were presented with masks at both moments, in the study and test phase, results of the multiple regression showed a significant collective effect between the trustworthiness, dominance, and distinctiveness, and the recognition performance, *F*(3, 44) = 3.53, *p* = .02, *R*^2^ = .19, meaning that the three factors are related to face recognition. However, upon further examination, only the facial distinctiveness, *t*(44) = 2.28, *p* = .03, 95% CI [0.04, 0.60], and the trustworthiness, *t*(44) = −2.41, *p* = .02, 95% CI [−0.63, −0.06], were significant predictors in the model, meaning that facial distinctiveness and trustworthiness judgments are the only factors that can predict the face recognition performance.

The same pattern of results was obtained when faces were presented with masks during the study and without a mask in the test phase. The multiple regression indicated that a significant collective effect was found between the trustworthiness, dominance, and distinctiveness, and the recognition performance, *F*(3, 44) = 6.95, *p* < .001, *R*^2^ = .32, but upon further examination, only the facial distinctiveness, *t*(44) = 3.19, *p* = .003, 95% CI [0.18, 0.80], and the trustworthiness, *t*(44) = −3.56, *p* < .001, 95% CI [−0.76, −0.21], were a significant predictor in the model, meaning that facial distinctiveness and trustworthiness judgments are the only factors that can predict the face recognition performance.

Again, the same pattern of results was obtained when faces were present without a mask in the study phase but with a mask in the test phase. A significant collective effect was found between the trustworthiness, dominance, and distinctiveness and the recognition performance, *F*(3, 44) = 5.15, *p* = .004, *R*^2^ = .26, meaning that the three factors are indeed related to face recognition. However, only the facial distinctiveness, *t*(44) = 2.38, *p* = .02, 95% CI [0.05, 0.56], and the trustworthiness, *t*(44) = −2.75, *p* = .009, 95% CI [−0.68, −0.10], were significant predictors in the model, meaning that facial distinctiveness and trustworthiness judgments are the only factors that can predict the face recognition performance.

So, we can assume that when faces were presented with a mask at encoding, recognition, or both moments, the facial distinctiveness and the trustworthiness with which the faces are perceived in the study phase influence the posterior recognition performance. It is important to note that a higher facial distinctiveness and worse trustworthiness explain better face recognition when a mask is presented.

Finally, when the faces were presented without a mask in the two moments (i.e., the study and the test phase), results of the multiple regression indicated that a significant collective effect was found between the trustworthiness, dominance, and distinctiveness, and the recognition performance, *F*(3, 44) = 7.83, *p* < .001, *R*^2^ = .35, meaning that the three factors are indeed related to face recognition. However, upon further examination, only the facial distinctiveness, *t*(44) = 3.79, *p* < .001, 95% CI [0.22, 0.71] and the dominance, *t*(44) = −2.32, *p* = .03, 95% CI [−0.49, −0.03] were significant predictors in the model. These results mean that higher facial distinctiveness and lower dominance can explain better face recognition performance when the faces are presented without a mask.

## Discussion

With this study, we aimed to understand how masks impact face recognition and trait judgments and how trait judgments relate to face recognition of masked and unmasked faces. With this aim in mind, we conducted a within-participants experiment, where faces with and without masks were presented to participants. Besides replicating previous results (i.e., better memory for faces both presented unmasked in the study and the test phases), we expected to find enlightening data about the impact of masks on trait judgments (like trustworthiness, distinctiveness, and dominance) and how these traits judgments can affect face recognition of masked and unmasked faces.

### Effect of masks on face recognition

Confirming our first hypothesis, we observed a better recognition of faces shown without a mask during both phases. Another interesting result was the presence of a better recognition of faces encoded with a mask during the test phase. However, this result can be influenced by the masked–unmasked condition, where the worse face recognition occurred, confirming our second hypothesis. This was an expected result (Guerra et al., 2022; Manley et al., 2019) since, besides the affected holistic processing, a violation of the encoding specificity principle occurs in this case. In other words, the conditions presented in the study phase (i.e., masked faces) were disrupted in the test phase (i.e., unmasked faces), which led to a significant impairment of memory performance (Tulving & Thomson, 1973). In addition, in this condition (masked–unmasked faces), participants seem more conservative (i.e., higher values on response bias score—*c*), meaning that participants were likely more uncertain. So, they took fewer risks when recognizing faces.

This result, a more uncertain and worse memory for masked faces during the study but unmasked at the test phase, has significant implications, namely, in eyewitness testimony (e.g., Manley et al., 2019). A worse eyewitness identification will occur when the suspect is observed in the first moment with a mask and later—in the lineup—without a mask (which typically occurs). One possible alternative is to present the suspect (and fillers) at the lineup with a mask since face recognition was better for masked faces at the study and test phase than for masked faces at the study but unmasked in the test phase.

The encoding specificity principle was also violated in the unmasked–masked condition since the faces in the study phase were presented without masks. However, as referred to before, the holistic processing of the faces was allowed in this condition. Previous studies (Richler et al., 2011) have demonstrated that, even when faces might be submitted to certain deviations from their original form, the holistic processing of faces benefits their later recognition.

### Effect of masks on trait judgments

Regarding the effect of a mask on trait judgments, as postulated in our third hypothesis, we observe that unmasked faces were considered more distinctive. Since distinctiveness judgments are made mainly based on facial features, if these features were covered by a mask, less distinctive were the faces classified (Santos & Young, 2008). Also, we observed that masked faces were considered more trustworthy than unmasked faces, and this result aligns with previous studies (Oldmeadow & Koch, 2021; Olivera-La Rosa et al., 2020). One possible explanation was that the use of masks was an internalized social norm and, for this reason, led to higher trustworthiness judgments. However, it is important to note that using face masks is no longer mandatory. Another explanation is that trustworthiness was based on cues covered by the masks, so trustworthiness judgments increased.

Also, our fourth hypothesis was confirmed since no differences between masked and unmasked faces regarding dominance judgments were observed. This was an expected result since previous studies suggested that these judgments rely primarily on structural cues such as eyes-to-eyebrow distance and width-to-height ratio (Costa et al., 2017; Vernon et al., 2014), areas not affected using masks. Previous studies have also shown dominance impressions were less affected by masking than trustworthiness impressions (Oliveira & Garcia-Marques, 2022).

### Effect of trait judgments on face recognition

Finally, our main goal with this study was to observe how trait judgments affect the facial recognition of masked and unmasked faces. We can conclude that facial distinctiveness predicts face recognition, regardless of mask use. More distinctiveness of the faces leads to better face recognition. In contrast, dominance judgments only predicted face recognition when we presented faces without a mask in both moments (i.e., in the study and the test phase).

Therefore, when faces were presented with a mask at encoding, at recognition, or both moments, the dominance judgments seem no longer to explain face recognition’s performance. In this case, the trustworthiness judgments become a more important variable. Thus, we can conclude that when faces were presented with a mask, regardless of the phase (study or test phase), trustworthiness overrides dominance becoming more critical to recognizing faces, where faces considered untrustworthy were better recognized.

We can interpret these results from an evolutionary perspective. In personal encounters, people must determine the intentions of the other person or group - if they have good or bad intentions (Fiske et al., 2007). In the last years, social cognition studies established that people distinguished others by traits related to perceived intent (e.g., trustworthiness) and perceived ability (e.g., dominance). Although trustworthiness and dominance dimensions are both crucial variables, probably when a survival context was promoted (e.g., where masks were presented), the first variable became more relevant. Indeed, evidence suggests that the trustworthiness judgment carries more weight in affective and behavioral reactions because another person’s intent is more important to survival than whether the other person can act on those intentions (Fiske et al., 2007).

Nairne et al. (2007) have already shown that human memory systems have evolved to retain information relevant to the individual’s fitness. The authors observed that participants showed the best memory performance for words rated for survival scenario relevance. This memory advantage was consistent across recall and recognition memory tests for within- and between-participant designs (Nairne et al., 2007). So, it seems that processing information within a survival-relevant scenario might provide memory benefits for information.

Interestingly, in a study by Hou and Liu (2019), participants were asked to predict their avoidance or approach response tendencies when encountering strangers in a survival and control scenario. These strangers’ facial trustworthiness (untrustworthy, neutral, and trustworthy) was manipulated. Recognizing untrustworthy faces was significantly more accurate than trustworthy and neutral faces in the survival scenario, suggesting a significant negative bias in survival contexts. This result is consistent with previous studies showing that threatening information is easier to recognize and remember than neutral or positive information (Kensinger, 2007). The authors concluded that untrustworthy faces are more accurately recognized than trustworthy faces because negative events can help humans avoid potential threats (Hou & Liu, 2019).

Besides a better memory for perceived-untrustworthy faces (Rule et al., 2012), these faces are considered more relevant for survival (i.e., the functional account of memory) and highly salient (i.e., the functional account of perception). It can explain why, in this study, trustworthiness became a more relevant variable for faces presented with masks, creating a better memory for untrustworthy faces. Curiously, Rule et al. (2012) showed that although the influence of trustworthiness on face recognition can be manipulated by verbal information (e.g., Stole money from his [her] mother’s purse), the independent contribution of the face was statistically more significant.

### Limitations and future studies

Since it was previously observed an own-age bias, where participants recognized better faces of the same age group than different age groups (Rhodes & Anastasi, 2012), we decided to match the age of the presented faces (*M* = 23.82, *SD* = 1.81) with the age of the participants (*M* = 20.21, *SD* = 2.65). With this procedure, we controlled the influence of the own-age bias on face recognition. However, in future studies, the effect of own-age bias and possible interactions between age and the use of masks can be explored.

Besides age, another important variable to consider is the gender of the participants. It was previously observed that the trustworthiness judgments depend on the perceiver’s gender. Namely, women judge trustworthy-looking faces as significantly more trustworthy than men. This result was more pronounced for judgments of female faces (Mattarozzi et al., 2015). Given that 92% of our study sample consisted of female participants, future studies can also observe how the gender of the participants can influence the personality judgments and the face recognition of female and male (masked and unmasked) faces.

It is also important to note that some evidence has been raised that using highly similar images of the face in the two phases (i.e., study and test phases) cannot simply be considered a face recognition procedure since it can be viewed as a picture recognition one (Burton, 2013). However, our study used the same image of the face in both phases (i.e., in the study and test phase), specifically in the congruent conditions. Using the same image allowed us to control and eliminate variance in all dimensions except those we wish to manipulate—the presence of face masks.

Also, it is important to note that the same image was presented in our congruent conditions (masked at the study and the test phases and unmasked at the study and the test phases). In contrast, in incongruent conditions, the image has a slight change: the presence of a mask. It was previously observed that it is easier to recognize pictures than faces (Burton, 2013), so maybe better memory for the congruent conditions occurred because it is the exact same image. However, considering our results, the memory in the condition unmasked at the study—masked at the test was better than in the condition masked at the study—masked at the test. So, we did not observe a better memory when participants were presented with the same images compared with the presentation of images slightly different. Nevertheless, it will be interesting if future studies replicate our study but use different photos of the same people. For example, taken in the same photographic session and with the same camera, but with a change of pose.

In sum, our results reveal that using a face mask hampers face recognition, mainly if used in the encoding phase but not present in the test phase. As referred before, in the legal settings, the conditions (e.g., the use of masks) between the study and the test phase should be matched if possible. Also, this experiment let us conclude that if holistic processing is allowed during encoding, the detrimental effects caused by mask use on face recognition can be mitigated. So, we can assume that more important than the later moment (the test phase), the conditions presented in the first contact (the study phase) are really important. If we meet someone who does not use a mask, it will be easier to recognize this person later since we hold previous information of a “complete face version” (Guerra et al., 2022).

Finally, we established that the use of masks impacts some trait judgments (like trustworthiness and distinctiveness but not dominance), being that in the presence of masks, the trustworthiness with which the faces are perceived is a significant predictor of face recognition. So, when someone uses a mask, the trustworthiness evaluation that we make about this person is a significant predictor of their recognition. If we considered a masked person as not trustworthy at all, we would probably recognize better this person latter.

## Data Availability

https://osf.io/5mjkv/?view_only=06e45ed1d0064cb0b86a9a96f2675899.
